# TGF-beta promotes human T follicular helper cell stemness properties at the expense of effector function

**DOI:** 10.3389/fimmu.2026.1732275

**Published:** 2026-03-25

**Authors:** Yixin Yang, Chanfeng Wu, Wenjin Yang, Lanxin Peng, Yunhao Zhang, Xingyu Zheng, You Wang, Kun Jin, Wenpei Liu

**Affiliations:** 1 College of Basic Medical Sciences, Hengyang Medical School, University of South China, Hengyang, China; 2 Translational Medicine Institute, The First People’s Hospital of Chenzhou, Hengyang Medical School, University of South China, Chenzhou, China

**Keywords:** cell differentiation, effector function, stemness properties, Tfh (follicular helper T cells), TGF-beta

## Abstract

**Introduction:**

Follicular helper T (T_FH_) cells are essential for germinal center reactions and the maintenance of long-lived humoral immunity. Transforming growth factor-β (TGF-β) is a multifunctional cytokine implicated in immune regulation, T-cell differentiation, and the maintenance of cellular stemness. Prior studies have shown that TGF-β promotes stemness across a wide range of cell types and facilitates the differentiation of naïve CD4⁺ T cells into various T helper cell subsets. However, its precise effects on T_FH_ effector function and stem-like properties remain poorly understood.

**Methods:**

The dual regulatory roles of TGF-β1 in modulating T_FH_ effector functions and stem-like properties were investigated using flow cytometry-based phenotyping, co-culture assays with memory B cells, proliferation and apoptosis assays, ELISA for antibody production, and bulk RNA sequencing of naïve-derived and blood-derived T_FH_ cells.

**Results:**

We found that TGF-β1 treatment in vitro promoted human naïve CD4^+^ T cells differentiation into CXCR3^+^ TFH, but significantly attenuated their effector molecule expression and T_FH_-mediated memory B-cell differentiation and antibody production, whereas it enhanced the expression of stemness-associated molecules in T_FH_ cells both differentiated in vitro from naïve CD4^+^ T cells and isolated from blood. Notably, TGF-β1 promoted proliferation and reduced apoptosis of naïve-derived T_FH_ cells in vitro, but suppressed proliferation and increased early apoptosis in blood-derived mature T_FH_ cells.

**Discussion:**

Our findings indicate that TGF-β1 tunes the balance between T_FH_ effector function and stem-like properties, and show differential regulations of the early phase of T_FH_ differentiation and mature T_FH_ cells, which may have implications for T_FH_-driven immune pathology and disease.

## Introduction

Follicular helper T (T_FH_) cells are a distinct subset of CD4^+^ T cells that play a central role in orchestrating the adaptive immune response. Specifically, they support B cell proliferation, maturation, and antibody production within the germinal centers of secondary lymphoid organs (1, 2). These cells were initially identified by their expression of CXCR5, which allows them to efficiently migrate into B cell follicles and facilitate effective interactions with B cells (3–5). T_FH_ cells perform their functions primarily through the secretion of specific cytokines, such as IL-21, and by engaging with B cell surface molecules, including CD40L and CD40 (6, 7). A comprehensive understanding of the mechanisms that regulate T_FH_ cell differentiation and function is essential for advancing our knowledge of immune responses and for the development of effective therapeutic interventions and vaccines.

Transforming growth factor-beta (TGF-β) is a pleiotropic cytokine that exerts diverse effects on cellular proliferation, differentiation, apoptosis, and immune regulation (8–10). Extensive research has explored its roles in immune cell function, with particular attention to its impact on T_FH_ cells. TGF-β has been shown to modulate T_FH_ cell responses, promote a regulatory phenotype, and influence autoantibody production in autoimmune diseases (11, 12). Moreover, during immune responses to pathogens, TGF-β plays a crucial role in shaping the immune landscape by directing the differentiation of naïve CD4^+^ T cells into T_FH_ cells through the regulation of key transcription factors such as BCL6 and c-MAF (13, 14). Conversely, IL-2-mediated signaling inhibits T_FH_ differentiation (15, 16), and whether TGF-β promotes human T_FH_ differentiation, at least partly, by limiting this pathway also needs to be confirmed.

Numerous studies have demonstrated that TGF-β is instrumental in inducing stemness properties across various cell types. It promotes self-renewal in stem cells by inhibiting differentiation, thereby preserving the stem cell pool (17, 18). Furthermore, TGF-β has been shown to maintain stemness features in multiple biological contexts, including tumor development, hematopoiesis, and immune regulation (19–21). However, under chronic antigen exposure, sustained TGF-β1 signaling is more closely linked to reinforcing CD8^+^ T-cell exhaustion than to preserving stem-like potential. Evidence suggests that SMAD2/3-dependent transcription and epigenetic remodeling contribute to stabilizing terminal exhaustion and reducing reinvigoration in response to immune checkpoint blockade (22, 23). Considering the critical roles of T_FH_ cells in immune responses, it is therefore important to investigate whether TGF-β promotes stemness-associated features in T_FH_ cells.

In this study, we investigated the functional characteristics and stemness properties of naïve-derived and memory T_FH_ cells in response to TGF-β1 treatment. Through a combination of cell culture experiments, flow cytometry, and bulk RNA sequencing, we demonstrate that TGF-β1 enhances stemness-associated transcriptional and phenotypic features in both naïve CD4^+^ T cell-derived T_FH_ cells and circulating memory T_FH_ cells, while simultaneously attenuating their effector functions.

## Result

### TGF-β1 promotes human naïve CD4^+^ T cells differentiation into CXCR3^+^ T_FH_ cells

**Figure 1:**
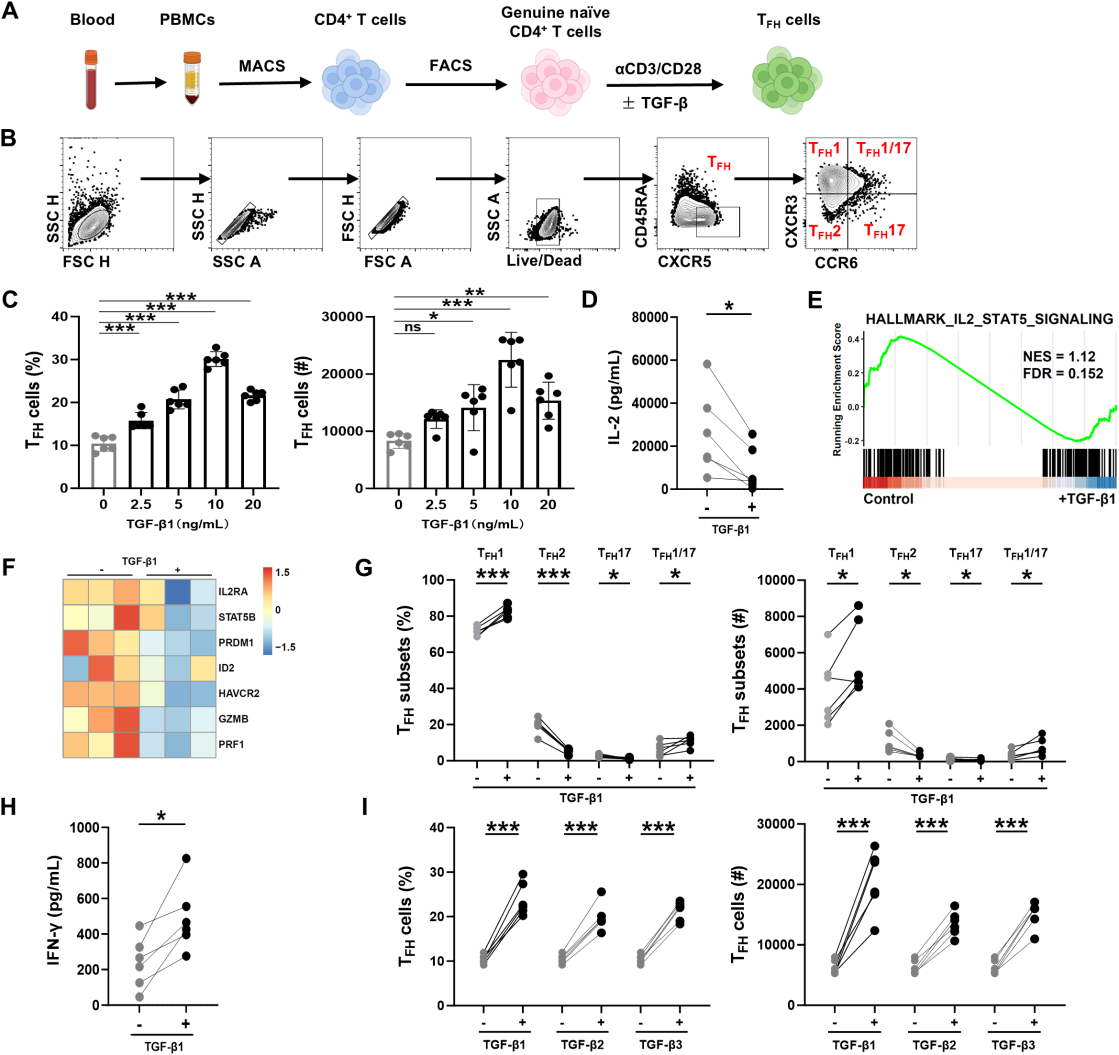
TGF-β1 promotes human naïve CD4+ T cells differentiation into CXCR3^+^ T_FH_ cells. **(A)** Schematic of T_FH_ cell differentiation assay. Peripheral blood mononuclear cells (PBMCs) were isolated from healthy donors, and naïve CD4^+^ T cells (CD4^+^ CD45RA^+^ CCR7^+^ CD95^-^ CD58^-^) were purified and stimulated *in vitro* with anti-CD3/CD28 beads ± TGF-β1. **(B)** Gating strategy for T_FH_ cells (CD4^+^ CD45RA^-^ CXCR5^+^) and subsets based on CXCR3/CCR6: T_FH_1 (CXCR3^+^ CCR6^-^), T_FH_2 (CXCR3^-^ CCR6^-^), T_FH_17 (CXCR3^-^ CCR6^+^), and T_FH_1/17 (CXCR3^+^ CCR6^+^). **(C)** TGF-β1 dose-response analysis. Purified naïve CD4^+^ T cells were stimulated with increasing concentrations of TGF-β1 (0, 2.5, 5, 10, and 20 ng/mL). The gating was performed according to **(B)**. Bar graphs show T_FH_ cell frequencies and absolute counts (mean ± SD; n = 6 donors). Statistical comparison to 0 ng/mL: one-way ANOVA. **(D)** IL-2 levels in culture supernatants collected on day 3 from naïve CD4^+^ T cells stimulated with anti-CD3/CD28 beads in the absence or presence of TGF-β1 (10 ng/mL), as quantified by ELISA (n = 6). **(E)** Gene Set Enrichment Analysis (GSEA) of RNA-seq profiles from differentiated T_FH_ cells (control vs. TGF-β1-treated), based on the HALLMARK_IL2_STAT5_SIGNALING gene set (NES = 1.12, FDR = 0.152). **(F)** Heatmap showing normalized expression of representative genes in the IL-2-STAT5 pathway across individual donors in control and TGF-β1-treated T_FH_ cells. **(G)** Subset-specific TGF-β1 (10 ng/mL). Frequencies and absolute counts of T_FH_ subsets were analyzed. Data represent mean ± SD (n = 6 donors). Statistica: paired t-test per subset vs. untreated. **(H)** IFN-γ levels in culture supernatants collected on day 3 from the same cultures as in **(D)**, as quantified by ELISA (n = 6). **(I)** Comparative effects of TGF-β isoforms (10 ng/mL). All isoforms increased T_FH_ frequencies/counts similarly (mean ± SD; n = 6 donors). Statistics: paired t-test vs. 0 ng/mL. Each symbol represents one donor with paired samples connected by lines. Significance thresholds: *P < 0.05; **P < 0.01; ***P < 0.001. P < 0.05 was considered to be a two-tailed significant difference, ns, not significant.

To examine the impact of TGF-β on T_FH_ cell differentiation, peripheral blood mononuclear cells (PBMCs) were isolated from healthy donors. To exclude the rare population of stem cell-like T cells displaying a naïve phenotype (24, 25), genuine naïve CD4^+^ T cells (CD3^+^ CD4^+^ CD45RA^+^ CCR7^+^ CD95^-^ CD58^-^) were enriched using magnetic beads and subsequently sorted by flow cytometry. Sorted naïve cells were stimulated with anti-human CD3/CD28 beads for 3 days in the presence of various doses of TGF-β1. T_FH_ cells (CD4^+^ CD45RA^-^ CXCR5^+^) and their subsets were analyzed by flow cytometry after stimulation (**Figures 1A, B**). We found that both the proportions and absolute cell numbers of T_FH_ cells derived from naïve CD4^+^ T cells were increased in the presence of TGF-β1 from 0 to 10 ng/mL in a dose-dependent manner, while declined in the dose of 20 ng/mL (**Figure 1C**). These data confirm that a low dose of TGF-β1 efficiently promotes human T_FH_ cell differentiation *in vitro*. However, whether this effect is accompanied by suppression of established negative regulatory pathways, such as the IL-2-STAT5 axis, remains to be determined. Therefore, we first assessed IL-2 secretion levels in the culture supernatant by ELISA to evaluate whether TGF-β1 affects this negative regulator, and found that IL-2 levels decreased following TGF-β1 stimulation (**Figure 1D**). As IL-2 is a key inducer of the STAT5 pathway, the reduction in supernatant IL-2 suggests that TGF-β1 may further attenuate the IL-2-STAT5 signaling pathway. To directly verify this, T_FH_ cells differentiated from naïve CD4^+^ T cells stimulated with anti-human CD3/CD28 beads, either in the absence or presence of TGF-β1, were sorted by flow cytometry, and RNA sequencing was performed on these cells. Subsequently, we analyzed pathway activity using GSEA and found that the IL-2-STAT5 pathway was suppressed following TGF-β1 treatment, with the heatmap showing downregulation of key genes in the pathway, such as IL2RA, STAT5B, PRDM1, and GZMB (**Figures 1E, F**). In summary, TGF-β1 treatment not only reduces IL-2 secretion in the culture system but also suppresses IL-2-STAT5 signaling at the transcriptomic level and downregulates its key genes, supporting a model in which TGF-β1 promotes human T_FH_ differentiation by at least partially antagonizing the IL-2-STAT5 axis.

Human circulating T_FH_ cells can be classified into several subsets based on the expression of CXCR3 and CCR6 (26, 27). Here we found that TGF-β1 (10 ng/mL) treatment mainly promotes the generation of CXCR3^+^ T_FH_ (including T_FH_1 and T_FH_1/17) while significantly inhibits the differentiation of CXCR3^-^ T_FH_ cell (includingT_FH_2 and T_FH_17) (**Figure 1G**). To further evaluate the cytokine profiles associated with subset polarization, IFN-γ levels in the supernatants were measured using ELISA (**Figure 1H**). The results showed an increasing trend in IFN-γ levels in the supernatants following TGF-β1 stimulation, further indicating that TGF-β1 shifts the cell population towards a T_FH_1-like phenotype. The TGF-β family comprises three isoforms-TGF-β1, TGF-β2, and TGF-β3, which are conserved in mammals (28). Although these isoforms share structural and signaling features, they exhibit distinct, context-dependent biological functions (28, 29). To assess the effects of different TGF-β isoforms on T_FH_ differentiation, naïve CD4^+^ T cells were stimulated with anti-human CD3/CD28 beads in the presence of an equal concentration (10 ng/mL) of TGF-β1, TGF-β2, or TGF-β3. All three isoforms promote human T_FH_ cell differentiation in a similar manner (**Figure 1I**). In summary, TGF-β1 promotes the differentiation of human naïve CD4^+^ T cells into T_FH_ cells, at least in part, by suppressing IL-2-STAT5 signaling, and it preferentially drives CXCR3^+^ T_FH_ differentiation.

### TGF-β1 inhibits the effector function of naïve-derived T_FH_ cells

**Figure 2:**
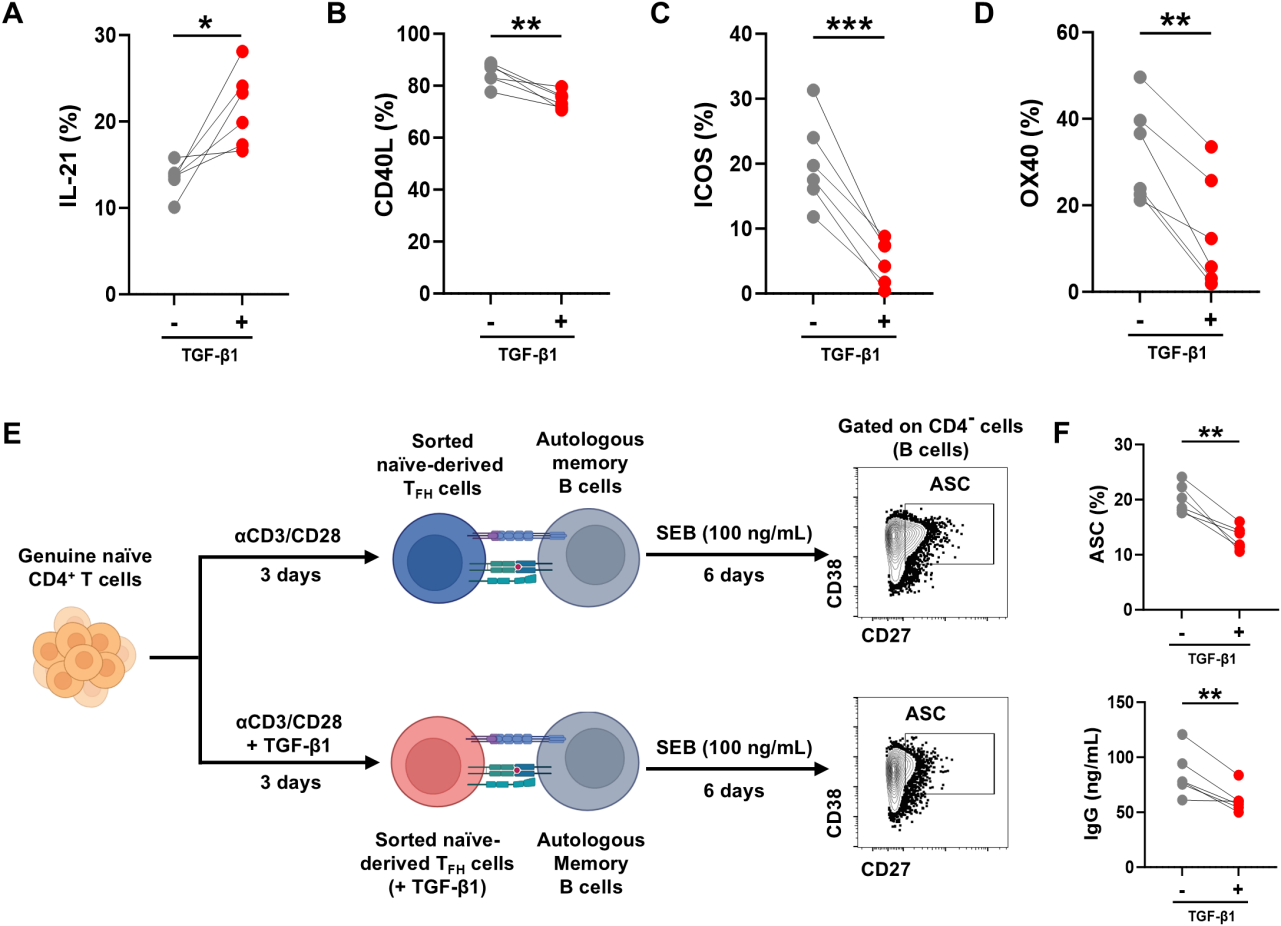
TGF-β1 inhibits effector function of naïve-derived T_FH_ cell. **(A-D)** Increased IL-21 production but reduced surface expression of CD40L, ICOS, and OX40 in T_FH_ cells stimulated with TGF-β1 (10 ng/mL) versus untreated controls (n = 6). **(E)** Schematic diagram of the TGF-β1-pretreated or untreated naïve-derived T_FH_ cells co-culture with autologous memory B cells in the presence or absence of TGF-β1. After co-culture, antibody-secreting cell (ASC; CD4^-^ CD19^+^ CD27^+^ CD38^+^) were analyzed by FACS, and the IgG concentration in the coculture supernatants was measured by ELISA. **(F)** Comparison of the frequencies of ASC frequency and IgG concentration in supernatants (n = 6). Statistics: paired t-test vs. untreated controls. Significance thresholds: *P < 0.05; **P < 0.01; ***P < 0.001. P < 0.05 was considered to be a two-tailed significant difference.

Although TGF-β promotes human naïve CD4^+^ T cell differentiation into T_FH_ cells, whether TGF-β affects the T_FH_ cell function remains to be further explored. T_FH_ cells are characterized by the expression of costimulatory molecules such as ICOS, CD40L, and OX40, and by the secretion of the signature cytokine IL-21, which together are essential for supporting B-cell activation, class switching, and the production of high-affinity antibodies (30, 31). To this end, the expression of CD40L, ICOS, OX40 and IL-21 in naïve-derived T_FH_ cells with or without TGF-β1 (10 ng/mL) was analyzed by flow cytometry. The surface expression of CD40L, ICOS and OX40 was markedly reduced after TGF-β1 treatment, whereas IL-21 production was increased (**Figures 2A–D**). Given that these T_FH_-associated molecules are critical mediators of B-cell help, the impact of TGF-β1 on the B-cell helper capacity of T_FH_ cells was further evaluated. First, sorted naïve CD4^+^ T cells were stimulated with anti-CD3/CD28 for 3 days in the presence or absence of TGF-β1. Subsequently 1×10^5^ naïve-derived T_FH_ cells were sorted by flow cytometry and co-cultured with equal numbers of autologous memory B cells for an additional 6 days in the presence of SEB (100 ng/mL) (**Figure 2E**). After co-culture, the frequencies of antibody-secreting cells (ASCs; CD4^-^ CD19^+^ CD27^+^ CD38^+^) and the IgG concentrations in culture supernatants were quantified by flow cytometry and ELISA, respectively. Lower proportions of ASCs and reduced IgG levels were observed when memory B cells were co-cultured with TGF-β1-pretreated T_FH_ cells compared with those co-cultured with untreated T_FH_ cells (**Figure 2F**).

Collectively, these findings indicate that although TGF-β1 promotes the differentiation of naïve CD4^+^ T cells into T_FH_ cells and enhances IL-21 expression, the expression of effector molecules such as CD40L, ICOS, and OX40 in T_FH_ cells was partially impaired by TGF-β1 treatment. Notably, the net impact on helper function of naïve-derived T_FH_ was attenuated by TGF-β1, as evidenced by reduced ASC differentiation and antibody production.

### TGF-β1 enhances stemness-associated gene expression, proliferation and survival of naïve-derived T_FH_ cells

**Figure 3:**
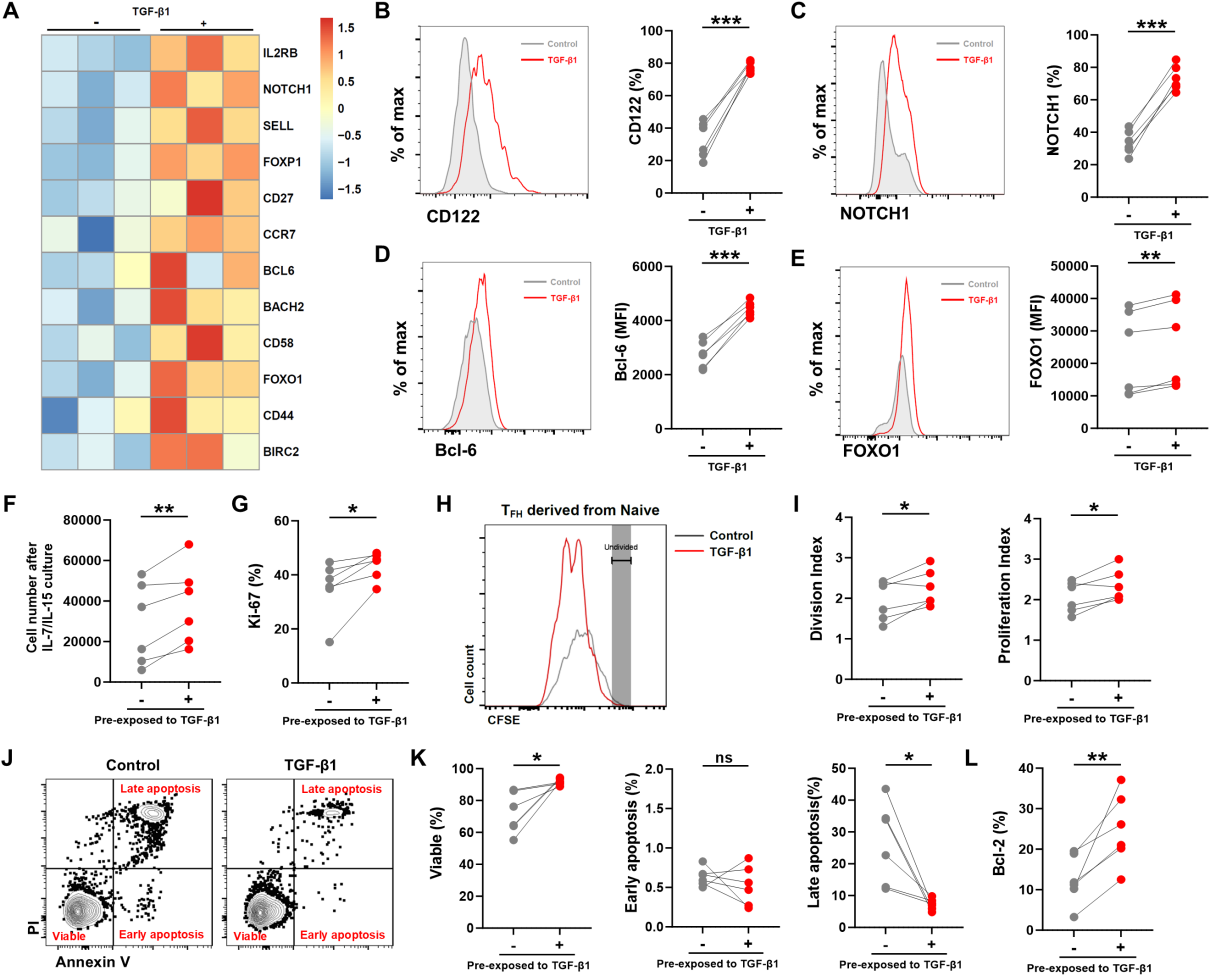
TGF-β1 upregulates markers associated with stemness while promoting proliferation and viability in naïve-derived T_FH_ cells. **(A)** Heatmap of stemness-associated genes and their expression in sorted naïve-derived T_FH_ cell populations. **(B-E)** Flow cytometric validation confirming increased protein expression: CD122 (IL2RB), NOTCH1, Bcl-6 (BCL6), and FOXO1 levels (quantified by mean fluorescence intensity, MFI) in naïve-derived T_FH_ cells following TGF-β1 treatment compared to untreated cells; histogram overlays (left) and MFI quantification (right) (n = 6). **(F-I)** Naïve CD4^+^ T cells were stimulated with anti-CD3/CD28 beads in the absence or presence of TGF-β1 (10 ng/mL) for 3 days to generate T_FH_ cells. T_FH_ cells were then sorted by flow cytometry, and equal numbers of sorted T_FH_ cells from each condition were re-plated and cultured with IL-7 and IL-15 for an additional 7 days before analysis. Total cell number after cytokine culture **(F)**, frequency of Ki-67^+^ cells **(G)**, CFSE dilution profile **(H)** and division/proliferation indices **(I)** were assessed at the end of the IL-7/IL-15 culture (n = 6). **(J, K)** Apoptosis was analyzed by Annexin V/PI staining after IL-7/IL-15 culture **(J)** and quantified as indicated **(K)** (n = 6). **(L)** Bcl-2 expression was measured by flow cytometry after IL-7/IL-15 culture (n = 6). Statistics: paired t-test vs. untreated controls. Significance thresholds: *P < 0.05; **P < 0.01; ***P < 0.001. P < 0.05 was considered to be a two-tailed significant difference, ns, not significant.

It has been reported that TGF-β signaling promotes T-cell longevity and recall capacity by increasing their stemness (21, 32, 33). Here, we confirmed that TGF-β supports human naïve CD4^+^ T cell differentiation into T_FH_ cells *in vitro*, particularly favoring the generation of CXCR3^+^ T_FH_ cells. Concurrently, TGF-β1 treatment impairs their helper function by downregulating effector-associated molecules. However, whether TGF-β modulates the T_FH_ cell stemness-like properties remains undefined. To this end, we analyzed the RNA sequencing data from naïve-derived T_FH_ cells in the presence and absence of TGF-β1. The RNA-seq results revealed that T_FH_ cells derived from naïve CD4^+^ T cells stimulated with TGF-β1, compared with those without TGF-β1 treatment, exhibited increased transcription of genes associated with T-cell stemness and memory potential, including *IL2RB*, *SELL*, *NOTCH1*, *BCL6*, *FOXO1* and *FOXP1* (**Figure 3A**). Partial gene expression such as CD122, NOTCH1, BCL6, and FOXO1 was validated by flow cytometry (**Figures 3B–E**). These data suggest that TGF-β1 enhances T_FH_ cell stemness and memory potential by upregulating stemness or memory associated gene expression.

To determine whether the TGF-β1-imprinted program translates into functional advantages during the maintenance phase, T_FH_ cells were first generated from naïve CD4^+^ T cells by anti-CD3/CD28 stimulation in the absence or presence of TGF-β1. After 3 days, T_FH_ cells were sorted by flow cytometry, and equal numbers of cells from each condition were then replated and cultured with the homeostatic cytokines IL-7 and IL-15 for an additional 7 days before analysis. Under these cytokine-driven conditions, T_FH_ cells previously exposed to TGF-β1 yielded significantly higher cell numbers after culture (**Figure 3F**), indicating enhanced homeostatic expansion from the same starting input. Consistently, TGF-β1-conditioned T_FH_ cells showed a higher fraction of Ki-67^+^ cells and increased proliferative expansion (**Figures 3G–I**).

Consistent with the increased cell recovery, Annexin V/PI staining after IL-7/IL-15 culture showed improved survival in the TGF-β1 group, reflected by a higher frequency of viable cells (**Figures 3J, K**). Early apoptosis was not significantly altered, whereas late apoptosis was reduced (**Figure 3K**). In line with the reduced late apoptosis, TGF-β1-conditioned T_FH_ cells exhibited an increased frequency of Bcl-2^+^ cells (**Figure 3L**), supporting a pro-survival effect during cytokine-mediated homeostatic maintenance. Collectively, these data indicate that TGF-β1 exposure during the initial differentiation phase endows naïve-derived T_FH_ cells with enhanced proliferative capacity and survival during subsequent antigen-independent homeostatic proliferation.

### TGF-β1 also suppresses the B cell helper function of circulating mature memory T_FH_ cells

**Figure 4:**
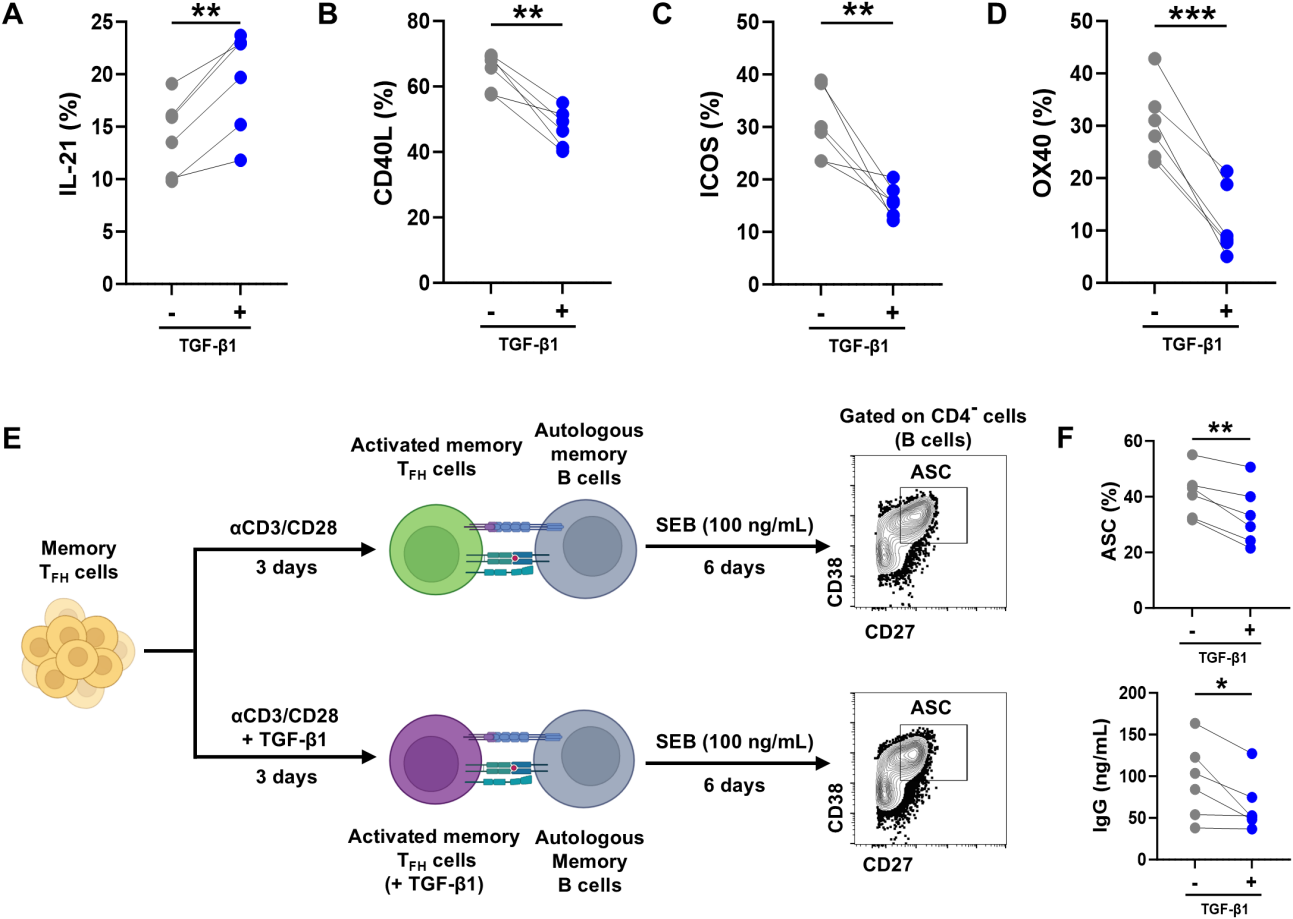
TGF-β1 inhibits effector and helper functions of circulating memory T_FH_ cells. **(A-D)** Surface expression of ICOS, CD40L, OX40, and intracellular IL-21 production in circulating memory T_FH_ cells isolated from PBMCs and stimulated for 3 days with anti-CD3/CD28 beads ± TGF-β1 (10 ng/mL), showing reduced costimulatory molecules but increased IL-21 (n = 6). **(E)** Schematic diagram: Memory T_FH_ cells pretreated with anti-CD3/CD28 beads ± TGF-β1 co-cultured with autologous memory B cells. After co-culture, antibody-secreting cell (ASC; CD4^-^ CD19^+^ CD27^+^ CD38^+^) were analyzed by FACS, and the IgG concentration in the coculture supernatants was measured by ELISA. **(F)** Comparison of the frequencies of ASC frequency and IgG concentration in supernatants (n = 6). Statistics: paired t-test vs. untreated controls. Significance thresholds: *P < 0.05; **P < 0.01; ***P < 0.001. P < 0.05 was considered to be a two-tailed significant difference.

Memory T_FH_ cells have undergone antigen-driven selection, differentiation, and maturation *in vivo*, and they exhibit robust B cell helper functions (26, 34). To examine the impact of TGF-β1 on memory T_FH_ cells, circulating memory T_FH_ cells were isolated from PBMCs, stimulated with anti-human CD3/CD28 beads for 3 days in the absence or presence of TGF-β1. After stimulation, the surface expression of ICOS, CD40L and OX40, as well as intracellular IL-21 production in T_FH_ cells, were analyzed by flow cytometry. The results showed that the surface expression of ICOS, CD40L, and OX40 in T_FH_ cells was markedly reduced, whereas IL-21 production was significantly increased after TGF-β1 treatment (**Figures 4A–D**), exhibiting a pattern similar to that observed in naïve-derived T_FH_ cells. To further evaluate the net impact of TGF-β1 on mature T_FH_ helper function, a co-culture experiment between memory T_FH_ cells and autologous memory B cells was performed. The results showed that TGF-β1 pretreatment significantly impaired T_FH_ cell helper function, resulting in reduced ASC differentiation and IgG production (**Figures 4E, F**). These results suggest that TGF-β1 treatment also impairs the effector and helper functions of memory T_FH_ cells, consistent with the observations in naïve-derived T_FH_ cells.

### TGF-β1 enhances stem-like properties while suppresses proliferation and induces early apoptosis in circulating memory T_FH_ cells

**Figure 5:**
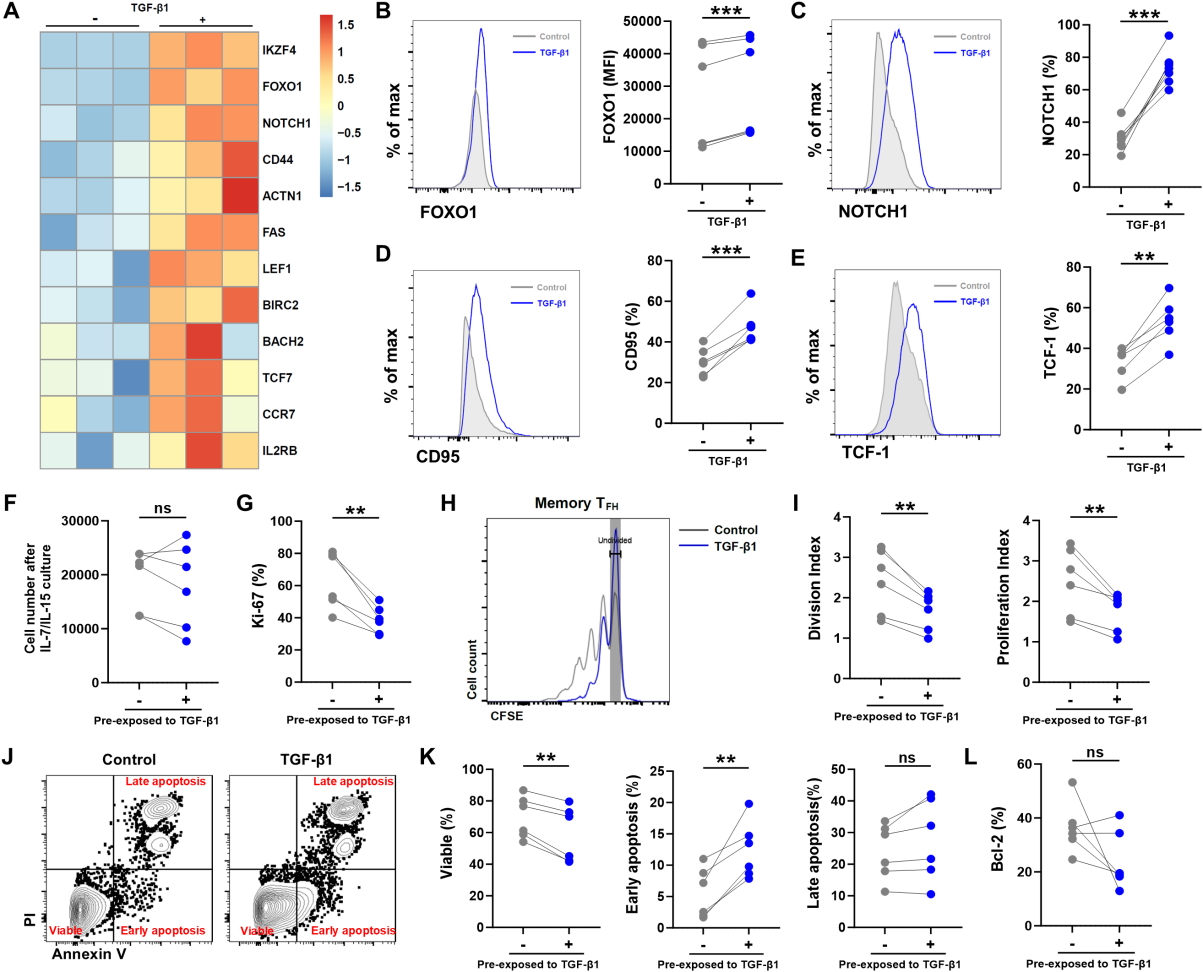
TGF-β1 regulates stem-like characteristics but inhibiting proliferation and promoting early apoptosis in circulating memory T_FH_ cells. **(A)** Heatmap showing upregulated transcription of stemness/memory-associated genes in flow-sorted circulating memory T_FH_ cells stimulated 3 days with anti-CD3/CD28 beads ± TGF-β1 (10 ng/mL) versus untreated counterparts. **(B-E)** Flow cytometric validation confirming increased protein expression of NOTCH1, FOXO1, CD95 (FAS), and TCF1 after TGF-β1 treatment: Histogram overlays (left) and mean fluorescence intensity (MFI) quantification (right) comparing TGF-β1-stimulated memory T_FH_ cells to unstimulated controls (n = 6). **(F-I)** After the 3-day stimulation, equal numbers of cells from each condition were replated and maintained with IL-7 and IL-15 for 7 days before analysis. Cell recovery **(F)**, Ki-67 frequency **(G)**, representative CFSE dilution **(H)**, and division/proliferation indices **(I)** are shown (n = 6). **(J, K)** Annexin V/PI analysis after IL-7/IL-15 maintenance with representative plots **(J)** and quantification of viable, early apoptotic, and late apoptotic fractions **(K)** (n = 6). **(L)** Bcl-2 expression after IL-7/IL-15 maintenance (n = 6). Statistics: paired t-test. **P < 0.01; ***P < 0.001. P < 0.05 was considered to be a two-tailed significant difference, ns, not significant.

Circulating memory T_FH_ cells are more differentiated than recently naïve-differentiated T_FH_ cells, but they still retain the potential for secondary responses and long-term maintenance (35). To determine whether TGF-β1 promotes stemness-related features in this subset, we analyzed the expression of stemness-associated surface markers and transcription factors in memory T_FH_ cells after TGF-β1 stimulation, following the same experimental approach as that used for naïve-derived T_FH_ cells. The results show that TGF-β1-stimulated memory T_FH_ cells also exhibited increased expression of genes linked to T-cell stemness and memory potential, including transcription factors *FOXO1*, *LEF1, TCF7*, as well as receptors/signaling molecules such as *NOTCH1*, *FAS*, and *CCR7* (**Figure 5A**), which are known to regulate T-cell survival, homeostatic proliferation, and tissue recirculation (36). Consistently, enhanced protein expression of NOTCH1, FOXO1, CD95, and TCF-1 was observed after TGF-β1 treatment, displaying a pattern similar to that seen in naïve-derived T_FH_ cells (**Figures 5B–E**).

To further align these stemness-associated gene changes with functional outputs, we next examined the expansion and survival status of circulating memory T_FH_ cells under the same stimulation conditions. Although the total recovered cell numbers were not significantly altered by TGF-β1 (**Figure 5F**), proliferative activity was markedly reduced, as indicated by a decreased frequency of Ki-67^+^ cells and diminished cell division in the presence of TGF-β1 (**Figures 5G–I**).

In parallel, Annexin V/PI staining revealed impaired survival upon TGF-β1 treatment. TGF-β1 decreased the proportion of viable cells and increased early apoptotic cells, whereas late apoptosis was not significantly changed (**Figures 5J, K**). Notably, the expression of the anti-apoptotic molecule Bcl-2 remained at comparable levels between groups (**Figure 5L**), suggesting that TGF-β1 primarily promotes an early apoptotic phenotype while restraining proliferative expansion in circulating memory T_FH_ cells. Overall, TGF-β1 induces a stemness- and memory-associated transcriptional and phenotypic program in both naïve-derived and circulating memory T_FH_ cells; however, the functional outcomes are context-dependent. Specifically, TGF-β1 conditioning enhances the proliferative capacity and survival of naïve-differentiated T_FH_ cells during IL-7/IL-15 maintenance, whereas it limits proliferative expansion and increases early apoptosis in mature memory T_FH_ cells.

## Discussion

In this study, we investigated the effects of TGF-β1 on the differentiation of human naïve CD4^+^ T cells into T_FH_ cells and their subsets, along with its impact on the effector function and stemness properties of both naïve-derived and circulating memory T_FH_ cells. TGF-β1 was found to promote T_FH_ differentiation, particularly that of the CXCR3^+^ subset, while attenuating T_FH_ effector and B cell helper function. By contrast, TGF-β1 enhanced stemness-related markers at both the transcriptomic and protein levels. However, its effects on proliferation and survival were cell-phase dependent: it promoted proliferation and survival during the early phase of T_FH_ cell differentiation, but constrained proliferative expansion and increased early apoptosis in mature circulating memory T_FH_ cells. These findings indicate that TGF-β serves as a bidirectional regulator, promoting human T follicular helper cell stemness features at the expense of their effector function, while exerting cell-phase dependent effects on proliferation and survival. This divergence may reflect differences in baseline epigenetic priming, activation history, or the balance of proliferative versus quiescence cues in these two T_FH_ compartments.

TGF-β is a multifaceted cytokine that has been implicated in immunosuppression and the maintenance of cellular stemness within the immune system (37–39). Previous research has shown that TGF-β1 co-opts with IL-12 or IL-23 promote T_FH_ cell differentiation via signaling STAT3-STAT4 signaling, which are implicated in autoimmune disorders, cancers, and chronic infections (14). Additionally, studies have shown that in T cells, the TGF-β-SMAD2 pathway exerts an antagonistic regulatory effect on the IL-2-STAT5 pathway (40). We confirmed that TGF-β inhibits the activation of this pathway, as reflected by the downregulation of IL-2 secretion, which may represent one of the mechanisms by which TGF-β promotes T_FH_ differentiation.

Understanding how TGF-β1 regulates T_FH_ function and stemness properties is crucial for elucidating disease mechanisms and for developing novel therapeutic strategies. In this study, we showed that a series of classical stemness-associated genes, such as SELL, FOXP1, and NOTCH1, were upregulated by TGF-β1. The upregulation of SELL and FOXP1 may reflect a shift toward a less differentiated, longevity-associated transcriptional state, as SELL has been linked to enhanced persistence and lymphoid-homing capacity of stem-like T cells (41), while FOXP1 functions as a key transcriptional regulator that preserves quiescence and restrains terminal differentiation in multiple T-cell contexts (42). Notably, NOTCH1 was among the most strongly induced genes at both the RNA and protein levels in TGF-β1-treated T_FH_ populations, suggesting that activation of this pathway may contribute to TGF-β1-induced plasticity and progenitor-like features of T_FH_ cells. Consistent with this observation, Notch signaling has been reported to be a central regulator of T-cell stemness and memory-like reprogramming (43, 44). In line with a direct role of Notch in T_FH_ biology, T cell-specific deletion of Notch1/2 in murine models has been reported to impair T_FH_ differentiation and germinal center B-cell responses (45). Moreover, cross-talk between the TGF-β and Notch pathways has been shown to contribute to maintaining progenitor-like T-cell states (46, 47). Taken together, these findings support a model in which TGF-β1 reinforces a stem/progenitor-like transcriptional program and plasticity in both naïve-derived and memory T_FH_ cells, at least in part via induction of Notch signaling, a pathway that is also required for T_FH_ differentiation and productive germinal center B-cell responses. However, the mechanisms by which TGF-β1 coordinates the regulation of Notch signaling remain to be elucidated.

In addition to direct effects on T cells, upstream myeloid cues may shape a T_FH_1-like trajectory that could synergize with TGF-β-dependent programming. GM-CSF-activated human CD1c^+^ DCs have been shown to drive naïve CD4^+^ T cells toward T_FH_1 differentiation in a CD40-dependent manner (48). It will be interesting to determine whether TGF-β-rich environments cooperate with such DC-derived polarization to reinforce (or reshape) the CXCR3^+^ T_FH_ state observed in our system.

The preferential induction of CXCR3^+^ (T_FH_1-like) T_FH_ cells may also have implications beyond humoral immunity, particularly in relation to CD8^+^ T-cell responses. In human breast cancer, functional T_H_1-oriented T_FH_ cells within TLS are associated with coordinated humoral and cytotoxic immune features, including granzyme-expressing CD8^+^ TILs (49). Consistently, T_FH_ cells can sustain CD8^+^-dependent antitumor immunity via IL-21, and TGF-β has been implicated in shaping a CXCL13 axis that facilitates T_FH_-CD8^+^ spatial coupling in tumors (50). These studies raise the possibility that a TGF-β-skewed T_FH_1-like program may coordinate antibody help with CD8^+^ T-cell immunity in specific inflammatory or tumor contexts.

Clinically, targeting TGF-β1 could help to prevent excessive humoral responses, thereby maintaining immune homeostasis and reducing the risk of autoimmune disease (51). In addition, given its established role in promoting stem-cell proliferation and differentiation, TGF-β1 may hold promise for stem-cell therapy and regenerative medicine (52). Moreover, by leveraging TGF-β1-mediated T_FH_ stemness, vaccines could be designed to maintain T_FH_ cells in a stem-like state, thereby enabling rapid responses upon antigen re-exposure, potentially through optimized antigen delivery and adjuvant selection (53).

TGF-β1 signaling is commonly divided into canonical SMAD-dependent and non-SMAD pathways. In the canonical pathway, TGF-β1 activates R-SMADs (e.g., SMAD2 and SMAD3), which form complexes with SMAD4 and translocate to the nucleus, where they regulate genes involved in self-renewal and differentiation, thereby maintaining stemness (54). In contrast, non-SMAD pathways including PI3K-AKT and RAS-MAPK promote stem-cell proliferation and survival, enhance self-renewal, and modulate inflammatory responses and stemness via NF-κB signaling (55, 56). TGF-β1 treatment led to marked transcriptional changes related to function and stemness in both naïve-derived and circulating memory T_FH_ cells. Targeting TGF-β1 signaling may enable therapeutic modulation of T_FH_ function in autoimmune disease and cancer, improve vaccine design for durable responses, and guide regenerative approaches that balance immune activity in autoimmune conditions.

Importantly, not all TGF-β superfamily ligands exert identical effects on T_FH_ function. Activin A has been identified as a potent regulator of human T_FH_-like differentiation and, in the presence of IL-12, can support a more complete phenotype including robust IL-21 potential (57). This contrasts with the context-dependent effects of TGF-β observed across systems and highlights that shared SMAD2/3 usage does not necessarily translate into shared functional outputs, particularly for key mediators such as IL-21. Dissecting how distinct TGF-β superfamily ligands integrate with the IL-2-STAT5 pathway and inflammatory cues may help explain divergent outcomes in T_FH_ helper function.

However, this study has several limitations. Experiments were primarily conducted *in vitro*, and additional *in vivo* studies are needed to define the effects of TGF-β1 on T_FH_ cells. Moreover, only transient effects of TGF-β1 on T_FH_-subset differentiation were examined. The long-term maintenance of T_FH_ phenotype, function and stem-like properties imprinted by TGF-β1 requires further investigated.

In conclusion, this study provides evidence that TGF-β1 promotes human naïve CD4^+^ T cells differentiation into CXCR3^+^ T_FH_ cells and enhances T_FH_ cell stemness-like properties at the expense of T_FH_ cell effector and B-cell helper functions. These findings provide a more comprehensive understandings of the role TGF-β1 in regulating T_FH_ cell biological properties, with underlying mechanisms worth further exploration.

## Materials and methods

### Human cell isolation and sorting

Peripheral blood mononuclear cells were purified from blood samples obtained from volunteers and informed consent was obtained from all the donors. Cell isolation and sorting were performed by magnetic bead and flow cytometry. Firstly, CD4^+^ T cells and CD19^+^ B cells were isolated from PBMCs by CD4 and CD19 microbeads (Miltenyi Biotec). The purified CD4^+^ T and B cells were further stained with Super Bright™ 600 anti-human CD4 (SK3, eBioscience), PE-eFluor 610 anti-human CXCR5 (MU5UBEE, eBioscience), PerCP/Cyanine5.5 anti-human CD45RA (HI100, BioLegend), FITC anti-human CCR7 (G043H7, BioLegend), PE-Cy7 anti-human CD95 (DX2, BioLegend), PE anti-human CD58 (TS2/9, BioLegend), and PE mouse anti-human CD20 (2H7, eBioscience), PE-Cy7 mouse anti-human CD27 (M-T271, BioLegend), respectively. Stained CD4^+^ T and B cells were sorted by FACS as follows: naïve CD4^+^ T cells (CD95^-^ CD58^-^ CD45RA^+^ CCR7^+^ CD4^+^ T cells), memory T_FH_ cells (CXCR5^+^ CD45RA^-^ CD4^+^ T cells), and memory B cells (CD27^+^ CD20^+^ CD19^+^ B cells).

### Stimulation of naïve CD4^+^ T cells and memory T_FH_ cells

Sorted naïve CD4^+^ T cells and memory T_FH_ cells were stimulated with anti-human CD3/CD28 Dynabeads (Invitrogen) in complete RPMI medium (RPMI-1640 medium containing penicillin-streptomycin, 50 µM 2-mercaptoethanol, 1 mM sodium pyruvate, nonessential amino acids and 25 mM HEPES, pH7.2-7.5) supplemented with 10% fetal bovine serum (FBS) in the absence or presence of the corresponding concentrations of recombinant human TGF-β1, or TGF-β2, or TGF-β3 (PeproTech) for 3 days. Naïve CD4^+^ T cell differentiation/effector function, memory T_FH_ cell effector function was measured by flow cytometry.

### Co-culture assay

Naïve CD4^+^ T cells were stimulated with anti-CD3/CD28 Dynabeads in the presence or absence of TGF-β1 for 3 days. Subsequently, naïve-derived T_FH_ cells were sorted by flow cytometry. A total of 1×10^5^ sorted T_FH_ cells were co-cultured with 1×10^5^ autologous memory B cells in complete medium for an additional 6 days. Where indicated, staphylococcal enterotoxin B (SEB) was added at 100 ng/mL. At the end of co-culture, antibody-secreting cells (ASCs) were identified by flow cytometry as CD4^-^ CD19^+^ CD27^+^ CD38^+^ cells. IgG concentrations in culture supernatants were measured by ELISA according to the manufacturer’s instructions. For experiments using circulating memory T_FH_ cells, purified memory T_FH_ cells were stimulated with anti-CD3/CD28 in the presence or absence of TGF-β1 for 3 days, after which viable cells were sorted and subjected to downstream analyses as indicated. Cells were isolated from six independent donors. Information regarding age and sex was not available.

### Homeostatic maintenance culture and proliferation/survival assays

After 3 days of stimulation, T_FH_ cells were sorted by flow cytometry from the corresponding cultures (for naïve-derived T_FH_) and from stimulated memory T_FH_ cultures (for memory T_FH_), based on the gating strategy described above. Importantly, equal numbers of sorted T_FH_ cells from each condition were re-plated at the same density and cultured in complete RPMI supplemented with IL-7 (10 ng/mL, KX-PROTEIN) and IL-15 (10 ng/mL, KX-PROTEIN) for an additional 7 days prior to analysis. Total cell numbers after cytokine culture were determined by flow cytometry. For proliferation analysis, sorted T_FH_ cells were labeled with CFSE/CellTrace prior to cytokine culture and CFSE dilution was analyzed after 7 days; division index and proliferation index were calculated in FlowJo (v10.8). For survival/apoptosis analysis, cells were stained with FITC Annexin V and propidium iodide (PI) after IL-7/IL-15 culture and the frequencies of viable, early apoptotic, and late apoptotic cells were quantified.

### Antibody staining and flow cytometry

Surface staining was performed for 30 minutes at 4 °C in phosphate-buffered saline containing 2% FBS. The fluorescently labelled antibodies used were as follows: Super Bright™ 600 anti-human CD4 (SK3, eBioscience), PerCP/Cyanine5.5 anti-human CD45RA (HI100, BioLegend), PE-eFluor 610 anti-human CXCR5 (MU5UBEE, eBioscience), PE anti-human CD40L (24-31, BioLegend), PE/Cy7 anti-human ICOS (C398.4A, BioLegend), APC/Cy7 anti-human OX40 (Ber-ACT35, BioLegend), PE anti-human CD38 (HB-7, BioLegend), PE/Cy7 anti-human CD27 (M-T271, BioLegend), Brilliant Violet 421™ anti-human CD122 (TU27, BioLegend), PE-Cy7 anti-human CD95 (DX2, BioLegend), FITC anti-human CCR7 (G043H7, BioLegend). To measure intracellular IL-21 secretion, cultured and purified T_FH_ cells were stimulated with phorbol 12-myristate 13-acetate (50 ng/mL) and ionomycin (1 μg/mL) for 6 h, brefeldin A (BioLegend) added during the final 4 h. For intracellular staining, cells were fixed and permeabilized using a fixation/permeabilization kit (BD Biosciences), followed by staining with PE anti-human IL-21 (3A3-N2, BioLegend), BV650 anti-human Notch1 (MHN1-519, BD Biosciences), PE-Cy7 anti-human Ki-67 (Ki-67, BioLegend), Alexa Fluor^®^ 488 anti-human Bcl-2 (100, BioLegend). For nuclear staining, cells were processed with the fixation/permeabilization concentrate (eBioscience), and subsequently stained with PE anti-human FOXO1 (W20064D, BioLegend), APC anti-human Bcl-6 (BCL-UP, eBioscience), Alexa Fluor^®^ 647 anti-human TCF-1 (7F11A10, BioLegend). The samples were acquired on a CytoFLEX SRT Flow Cytometer (Beckman Coulter) immediately after antibody staining. Gating strategies were established using fluorescence minus one (FMO) controls and unstained controls. Data were analyzed with FlowJo v10.8 software (TreeStar).

### Enzyme-linked immunosorbent assay

For IL-2 and IFN-γ quantification, culture supernatants were clarified by centrifugation when necessary and stored at ≤ -20 °C (or -80 °C) until analysis. IL-2 and IFN-γ levels were measured using the Human IL-2 and IFN-γ ELISA MAX™ Deluxe Set (BioLegend) following the manufacturer’s protocol. Briefly, 96-well plates were coated with diluted capture antibody overnight at 2-8 °C, washed, and blocked with Assay Diluent A for 1 h at room temperature (with shaking). Standards and appropriately diluted samples were then added for 2 h, followed by incubation with biotinylated detection antibody for 1 h and Avidin-HRP for 30 min. After washing, TMB substrate was added for color development, and the reaction was stopped with stop solution; absorbance was read at 450 nm within 15 min, and concentrations were calculated from the standard curve.

The concentration of IgG in the co-culture supernatants was determined using a human IgG ELISA kit (Mabtech) according to the manufacturer’s instructions. Briefly, 96-well plates pre-coated with anti-human IgG capture antibody were incubated with serially diluted IgG standards and appropriately diluted culture supernatant samples for 2 hours at room temperature. After washing, plates were incubated with HRP-conjugated anti-human IgG detection antibody, followed by the addition of TMB substrate solution. The colorimetric reaction was stopped with 2 N H_2_SO_4_, and absorbance was measured at 450 nm using a microplate reader. IgG concentrations were calculated based on the standard curve generated from known IgG concentrations.

### Bulk RNA-sequencing

Bulk RNA­seq was performed on 1×10^5^ purified naïve-derived T_FH_ cells and memory T_FH_ cells after cultured with anti-human CD3/CD28 beads in the presence or absence of 10 ng/mL TGF-β1 for 3 days. Purified T_FH_ cells were fully dissolved with 1 mL of Trizol (Takara) and sent to GENEWIZ (Suzhou, China) for library construction and sequencing analysis. Libraries were prepared using standard Illumina protocols and sequenced on a NovaSeq 6000 platform (Illumina) to generate 150 bp paired-end reads. Raw reads were processed using Cutadapt (v1.9.1) to remove adapter sequences and low-quality bases. Clean reads were aligned to the human reference genome (GRCh38) using HISAT2 (v2.2.1), and gene-level counts were quantified with HTSeq (v0.6.1). Further analyses were performed with R software (v4.4.1). Raw read counts were normalized, and differential expression was analyzed using the Bioconductor package DESeq2 (v1.6.3). Heatmaps were generated with the package pheatmap (v1.0.12). GSEA was calculated with the package ClusterProfiler (v4.12.6).

### Statistical analysis

Statistical analyses were performed using GraphPad Prism (version 10.1; GraphPad Software). Data are shown as mean ± SEM unless otherwise indicated. Comparisons between two groups were made using the paired two-tailed t-test, while multiple group comparisons were performed using one-way ANOVA followed by Tukey’s *post hoc* test. Statistical significance was defined as p < 0.05.

## Data Availability

RNA-seq data presented in the study are deposited in the GEO repository (https://www.ncbi.nlm.nih.gov/geo/), accession number GSE320556.
